# Styrene, (+)-trans-(1*R*,4*S*,5*S*)-4-Thujanol and Oxygenated Monoterpenes Related to Host Stress Elicit Strong Electrophysiological Responses in the Bark Beetle *Ips typographus*

**DOI:** 10.1007/s10886-019-01070-8

**Published:** 2019-05-04

**Authors:** Christian Schiebe, C. Rikard Unelius, Suresh Ganji, Muhammad Binyameen, Göran Birgersson, Fredrik Schlyter

**Affiliations:** 1 Chemical Ecology, Department Plant Protection Biology, Swedish University of Agricultural Sciences, PO Box 102, 230 53 Alnarp, Sweden; 2 Department Chemistry and Biomedical Sciences, Linnaeus University, Stuvaregatan 2, 392 31 Kalmar, Sweden; 3 Department of Entomology, Faculty of Agricultural Sciences & Technology, Bahauddin Zakariya University, Multan, 60800 Pakistan; 4 Faculty of Forestry & Wood Sci, Excellent Team for Mitigation, Czech University Life Sci Prague, Kamycka 129, Prague 6, 16521 Suchdol, Czech Republic

**Keywords:** Oxygenated monoterpenes, Styrene, 4-thujanol, GC-EAD, Single-sensillum recordings, SSR, Host chemistry, Plant defense

## Abstract

Bark beetles kill apparently vigorous conifers during epidemics by means of pheromone-mediated aggregation. During non-endemic conditions the beetles are limited to use trees with poor defense, like wind-thrown. To find olfactory cues that help beetles to distinguish between trees with strong or weak defense, we collected volatiles from the bark surface of healthy felled or standing *Picea abies* trees. Furthermore, living trees were treated with methyl jasmonate in order to induce defense responses. Volatiles were analyzed by combined gas chromatography and electroantennographic detection (GC-EAD) on *Ips typographus* antennae. Compounds eliciting antennal responses were characterized by single sensillum recording for identification of specific olfactory sensory neurons (OSN). Release of monoterpene hydrocarbons decreased, while oxygenated compounds increased, from spring to early summer in felled trees. In both beetle sexes particular strong EAD activity was elicited by trace amounts of terpene alcohols and ketones. 4-Thujanol gave a very strong response and the absolute configuration of the tested natural product was assigned to be (+)-*trans*-(1*R,*4*S,*5*S*)-thujanol by stereoselective synthesis and enantioselective gas chromatography. One type of OSN responded to all ketones and five other OSN were characterized by the type of compounds that elicited responses. Three new OSN classes were found. Of the eight EAD-active compounds found in methyl jasmonate-treated bark, the known anti-attractant 1,8-cineole was the one most strongly induced. Our data support the hypothesis that highly active oxygenated host volatiles could serve as positive or negative cues for host selection in *I. typographus* and in other bark beetles.

## Introduction

Hosts with weak or absent defenses, such as damaged or wind-broken trees, are the only choice for *Ips typographus* L. during long periods of low, “endemic”, population levels. Only when beetle populations increase into “epidemics”, and exceed a critical threshold can a mass-attack of beetles overcome the defense systems of healthy Norway spruce trees (*Picea abies* (L.) H. Karst.) (Mulock and Christiansen 1986). Host acceptance by the attacking bark beetles and resistance in healthy host trees are governed by the strength of induced defenses (Schiebe et al. 2012; Zhao et al. 2011). The ability to detect rare weakened hosts, still with sufficient nutritional quality but impaired defenses, is crucial for beetles in endemic populations, in order to avoid dispersal losses or death while attacking resistant host trees. At high beetle population densities, healthy hosts with high bark quality become colonizable. However, even during population outbreaks it is uncertain whether the first attacking pioneer beetles rely on sufficient numbers of conspecifics to overcome the defenses of strong-defending host trees, and thus avoid the threat to be killed. In the mountain pine beetle – lodgepole pine system (*Dendroctonus ponderosae* Hopkins *– Pinus contorta var. latifolia* Engelm.), trees with stronger defenses are preferred over trees with weaker defenses when beetle population levels are increasing (Boone et al. 2011; Raffa et al. 2016). In contrast to *Pinus* with high constitutive defenses, *P. abies* has weaker constitutive, but stronger induced defenses (Franceschi et al. 2005). Consequently, *I. typographus* beetles are probably more constrained to find weakened hosts and have to deal with unpredictable induced defenses encountered within the host, before a strong aggregation comes into effect. The same relationship was stressed by Raffa and Berryman (1987) for *Abies* spp. and *Scolytus ventralis* LeConte, when compared to *P. contorta* and* D. ponderosae*.

Attraction to aggregation pheromones is the most obvious natural behavior related to olfaction in *I. typographus* (Bakke et al. 1977; Schlyter and Birgersson 1999). Non-host volatiles (NHV), however, have been shown to have an antagonistic effect on the attractiveness of the pheromone (Unelius et al. 2014; Zhang and Schlyter 2004) and are a basis for the Semiochemical Diversity Hypothesis (Zhang and Schlyter 2003). Interference with long-range host finding by NHV has also been demonstrated (Schiebe et al. 2011). The proportions of olfactory sensory neurons (OSN) specific to pheromone, non-host, and host-related compounds are almost equal in *I. typographus* (Andersson et al. 2009), but little clear kairomonal attraction to host compounds alone has been shown (Schlyter and Birgersson 1999). In spite of ample electrophysiological data concerning host odor detection (Andersson 2012; Tømmerås and Mustaparta 1987), the importance of olfaction-based host choice by this insect is still a matter of debate (Baier and Bader 1997; Byers 1996; Gries et al. 1989; Tunset et al. 1993).

There is no evidence for kairomone-guided host attraction of *I. typographus* while numerous inhibitory cues for the beetle from both host and non-host plants have been reported (Andersson et al. 2010; Binyameen et al. 2014; Raffa et al. 2016; Zhang and Schlyter 2004). Andersson et al. (2009, 2010) reported the importance of some compounds that only occur in trace amounts in the host, as key ligands for OSNs. The response elicited by the cyclic ether 1,8-cineole inhibited the response in a co-localized OSN specifically tuned to the pheromone component *cis*-verbenol (Andersson et al. 2010). The 1,8-cineole could be found in significantly smaller amounts in trees that were susceptible to bark beetle attack than in trees that resisted attacks (Schiebe et al. 2012). Another trace constituent in host chemistry is the ketone verbenone eliciting strong responses from a specific OSN type. Microbial conversion of *cis-*verbenol and (−)-α-pinene to (−)-verbenone (Leufvén et al. 1984) gives rise to small amounts of (−)-verbenone in volatiles from trees under late stages of attacks (Birgersson and Bergström 1989; Byers et al. 1989). Verbenone has been shown to inhibit the attraction of *I. typographus* to its aggregation pheromone and might thus be a signal for an occupied, unsuitable host for beetles joining an ongoing attack (Schlyter et al. 1989) and is synergistic with NHV in inhibiting attraction (Unelius et al. 2014; Zhang and Schlyter 2003).

Like verbenone, other oxygenated monoterpenes in the bark of healthy trees are found only in trace amounts, but increase after bark beetle colonization (Leufvén and Birgersson 1987; Pettersson and Boland 2003). Induction of defense reactions with the phytohormone methyl jasmonate (MeJ) has been shown to result in a shift in the composition of volatiles emitted from Norway spruce foliage to a blend dominated by oxygenated monoterpenes and sesquiterpene hydrocarbons (Martin et al. 2003). So far, identification of oxygenated host compounds has not been included in studies concerning defense reactions in spruce bark (Erbilgin et al. 2006; Martin et al. 2002). The intricate balance between host defenses and beetle population dynamics, eventually resulting in successful colonization, provides an evolutionary pressure for sensory capabilities in bark beetles to evaluate host suitability by means of olfaction (Raffa et al. 2016). Later, after landing and during the initial feeding in the bark, non-volatiles (e.g., non-volatile lipids or phenolics) may provide gustatory cues to host acceptance (Faccoli and Schlyter 2007).

In this study we aimed to identify highly physiologically active trace constituents in the odor bouquet of *P. abies* bark; these might be missing links to understand how *I. typographus* could locate suitable host trees for colonization, either by attractive or by inhibitory cues. We examined volatile samples from intact bark surfaces from standing or felled trees with combined gas chromatography and electroantennographic detection (GC-EAD). EAD-active compounds were identified by combined gas chromatography-mass spectrometry (GC-MS) or synthesis and were further characterized by the single sensillum recording (SSR) technique. To understand how the production of these electrophysiologically-active trace volatile components is influenced by host-induced defense, we also analyzed bark tissue of trees locally treated with methyl jasmonate (Schiebe et al. 2012). Methyl jasmonate is a phytohormone known to elicit defense responses without inflicting physical damage (Krokene et al. 2008; Martin et al. 2002).

## Methods and Materials

### Experimental Site

Experimental trees were selected in February 2009 in a forest dominated by *P. abies* in Parismåla, SE Sweden (56° 35’ N, 15° 29′ E, 120–135 m a.s.l.). The selected trees were standing along the edges of clear-cuts where trees had been removed because of bark beetle attacks in 2007 and 2008.

### Collection of Bark Volatiles

Volatile samples were collected in parallel from the bark surface of cut trees at 50 cm from the root end, and in the middle of the tree crown. In standing trees, volatiles were collected either only at breast height or both at breast height and at approximately 5 m height. We avoided sampling from damaged bark or bark with obvious resin flow. Collection points were always placed on the side of the trunk exposed to the sun. Samples were collected from 13 April to 22 June 2009. A bent aluminum grid with open bottom area 29 × 10 cm and 3 cm height in the middle was fixed at the collection points to provide an open volume for volatile release. The aluminum cage was then wrapped with a double layer of 45 cm wide aluminum foil around the tree trunk, giving an open bark surface area for volatile release of approximately 5 dm^2^. The foil was closed at both ends with rubber straps and pressed against all irregularities of the bark to avoid holes towards the enclosure. The volatiles emanating from the bark surface within the enclosure were drawn through an adsorbent column placed in the open space of the enclosure, connected with a Teflon tube under the enclosure, and a silicon tube to a battery driven membrane pump. The collection column consisted of a Teflon tube (3 mm ID × 55 mm) filled with 35 mm (≈ 50 mg) Porapak Q® (50/80 mesh). The aeration columns were cleaned with 4 ml of dichloromethane and 5 ml of pentane prior to use. Before beginning collections, the air flow through each column was adjusted to 150 ml/min. Volatiles were always collected for 4 h. We assumed that the total volatile volume under the enclosure was exchanged every sixth minute and was trapped in the column, as no breakthrough was detected in a second column placed in line. Columns were also regularly placed outside the enclosure to record the background odor. The analyses of background odors never showed any antennally-active compounds except for the pinenes in spruce.

### Extraction and Analysis of Volatile Samples

Porapak columns were transported to the laboratory enclosed in glass vials and stored at −20 °C before solvent extraction. The adsorbed volatiles in each column were eluted with 1 ml of a 90:10 (*v*/v %) mixture pentane: diethyl ether with 40 μg heptyl acetate added as an internal quantification standard and collected in 1.5 ml glass vials. Prior to analyses, samples were concentrated to approximately 200 μl.

Volatile samples were analyzed as described in Schiebe et al. (2012) by combined gas chromatography and mass spectrometry (GC-MS) on a HP-5MS column. But with splitless injection and an initial oven temperature of 40 °C for 3 min, increased at 5 °C/min to 120 °C, held for 5 min at 120 °C followed by increase at 8 °C/min to 200 °C, and a final increase at 15 °C/min to 325 °C.

Compounds were identified by comparison of retention indices (van den Dool and Kratz 1963) and mass spectra with authentic standards, and with mass spectra in the Wiley library or Pherobase (El-Sayed 2018). Minor peaks were identified by ≥ three prominent ion fragments with overlapping ion chromatograms at the required retention time. These samples were later also analyzed by GC-EAD.

### Bark Sampling from Trees Induced by the Phytohormone Methyl Jasmonate (MeJ)

Two samples from undisturbed bark (constitutive defense) of seven trees were collected in April 2008, and on the same day the sampled trees were treated with MeJ to induce chemical defenses. This was achieved by removing a bark plug (9 mm diameter), placing a filter paper (9 × 9 × 0.5 mm) soaked with 50 mM MeJ on the exposed cambium surface and replacing the bark plug to seal the wound. One month later, two 1 × 2 cm samples of bark were taken above and below the MeJ treatment point. For a detailed description of MeJ-treatment and sampling, see Schiebe et al. (2012). Thus, we obtained two samples from each tree showing the bark chemical composition for both the constitutive and the induced state of the bark tissue.

The extraction followed the protocol described in detail by Schiebe et al. (2012) with few modifications. Thus frozen bark samples were ground in liquid nitrogen and submerged in 1.5 ml of the solvent mixture applied for headspace samples. In contrast to Schiebe et al. (2012), no measures were applied to remove polar components in the extracts in order to maximize recovery of oxygenated compounds. Analyses and quantification of the extracts followed the same GC-MS methodology as for volatile samples described above.

In measurements on trees under attack, the sampling area was kept free from beetles to avoid sampling from attacked and damaged bark. Colonisation of felled tree was recorded continuously and attack density was classified by estimation of visible attack entrances in four classes: (0) unattacked; (1) beginning colonisation with few entrance holes on entire tree; (2) scattered holes (<1 dm^−2^); (3) medium density (≈ 2–5 dm^−2^); and (4) maximum density (>5 dm^−2^).

### Quantification of MeJ Induced Metabolites in Trees

This procedure followed the protocol described in detail by Schiebe et al. (2012) with few modifications, but focused on compounds with GC-EAD activity. However, the peaks of some GC-EAD active compounds were too small and mixed with background noise to be quantified reliably with their total ion chromatograms (TIC). Thus, the peaks were based on extracted ion chromatograms of the base peak of their mass spectra and their proportion in TIC of authentic standards. Their amounts were adjusted to the peak area of the internal standard based in the same way on the proportion of its base peak to TIC. Quantification of 1,8-cineole was based on *m/z* = 139; 4-thujanol (sabinene hydrate) m*/z* = 71; heptyl acetate *m/z* = 43; camphor *m/z* = 108; pinocamphone *m/z* = 83; pinocarvone *m/z* = 81; 4-terpineol *m/z* = 71; α-terpineol *m/z* = 59; verbenone *m/z* = 107. Compounds were only quantified by their base peak when at least two more characteristic ion fragments co-eluted at the required retention time for compound verification. However, for 1,8-cineole we set our standard to have at least four ion fragments to be dissimilar from the co-eluted limonene and β-phellandrene mass spectra to allow identification and quantification by *m/z* = 139.

### Test Insects for Electrophysiology

Insects for electrophysiological studies were from laboratory-reared colonies or were collected from overwintering colonies on wind-felled logs in Parismåla, province Småland, Southern Sweden. Laboratory rearing conditions are described in Anderbrant et al. (1985). No differences were noticed in electrophysiological responses between beetles from different origins. Both sexes were used for all studies and sexes were separated by their external morphology (Schlyter and Cederholm 1981).

### Combined Gas Chromatography and Electroantennographic Detection (GC-EAD)

An Agilent 6890 GC (Agilent Technologies Santa Clara, CA, USA) and EAG apparatus (IDAC-2; Syntech, Kirchzarten, Germany) and were used for GC-EAD analyses. The GC was equipped with a fused silica capillary column (30 m × 0.25 mm) coated with either non-polar HP-5MS (film thickness = 0.25 μm; Agilent Technologies 19091S-433) or polar DB-Wax (film thickness = 0.25 μm; J&W 122–7032). Oven temperature was programmed for HP-5MS with an initial oven temperature of 50 °C for 3 min, increased at 5 °C/min to 150 °C, 3 min at 150 °C followed by 8 °C/min to 250 °C, and a final increase at 15 °C/min to 325 °C. For the DB-Wax column the temperature program was: initial temperature 30 °C for 3 min, increased at 5 °C/min to 80 °C, held for 3 min, 8 °C/min to 180 °C, and a final increase at 15 °C/min to 250 °C. Generally, 2 μl of volatile extracts or solutions of synthetic compounds were injected manually in splitless mode for 30 s before purge at an injector temperature of 225 °C.

Carrier gas was hydrogen at a linear flow of 45 cm s^−1^. Nitrogen (27.6 kPa, 4 psi) was added to the column effluent and this was then split 1:1 in a Gerstel 3D/2 low dead volume four way-cross (Gerstel, Mülheim, Germany) between the flame ionization detector (FID) and the EAD. The flow to the EAD passed through a Gerstel ODP-2 transfer line with 5 °C higher temperature than the GC oven and was led through a glass tube (ID 8 mm × 10 cm), and mixed with charcoal-filtered, humidified air at a flow rate of 0.6–0.9 l/min over the beetle antenna.

The *I. typographus* antennal preparations according to Zhang et al. (1999) were mounted as close as possible to the outlet of the glass tube. To verify electric potential drops as true responses to odorants, each volatile sample had to show simultaneous responses at the same retention time by at least four beetles. In addition, most verified responses were repeatedly detected in different samples. Recordings were obtained and assessed with the software GC-EAD versions 2009 1.1 and 2011 1.2.3 (Syntech).

All samples were run on HP-5MS and some were also run on DB-Wax in order to identify responses to compounds not separated on HP-5MS and to check that the retention times matched the reference compounds on both columns. For further identification of compounds eliciting responses, the same samples were run on GC-MS. Compounds repeatably eliciting EAD responses, and identified by GC-MS, were confirmed by repeated GC-EAD analyses with pure compounds or synthetic blends. The dose-response curves were obtained by running different concentrations of extracts in pentane.

### Test Compounds in Single Sensillum Recordings (SSR)

Compounds that elicited responses in GC-EAD recordings were further tested in SSR if available as synthetic references. A set of diagnostic compounds known as receptor ligands in previous studies was used for identification of OSN types (Table 2) (Andersson et al. 2009). We randomly selected 150 olfactory sensilla for screening among the two proximal undulating bands of sensilla. To save time we avoided sensilla in the distal part of the antennal club, as these house mainly OSNs tuned to the *I. typographus* pheromone component *cis*-verbenol and major host volatile α-pinene (Andersson et al. 2009).

All test compounds were diluted in paraffin oil (MERCK, Darmstadt, Germany). For screening, 10 μl of a 1 μg/μl dilution was applied on filter paper fitted in Pasteur pipettes, capped with plastic pipette tips. For dose-response tests, dilutions in 3–4 decadic orders of magnitude below the screening concentration were prepared and applied as above. Pipettes were used no longer than two days and stored at −20 °C (Andersson et al. 2009). Three compounds, styrene, (−)-β-pinene, and pinocarvone were tested only in a few recordings. Pinocarvone was tested as a concentrated resin extract with approximately 70% purity. When run by GC-EAD, only the main peak of this extract elicited any response and the extract was thus accepted as a test stimulus for pinocarvone in SSR. For 4-thujanol, the absolute configuration of the natural product tested was assigned to be (+)-*trans*-(1*R,*4*S,*5*S*)-thujanol by stereoselective synthesis and enantioselective gas chromatography, as detailed below in Results.

For each stimulus compound in dose-response tests, we used standard series of decadic steps as fixed doses on the filter paper, but later calculated these doses into corrected doses, giving the molar flux as the independent variable along the x-axis in plots to represent the best estimate of the stimuli reaching the preparation. These calculations compensate for differences both in molecular volatility and in affinity to paraffin oil (Andersson et al. 2012; de Fouchier et al. 2017).

### Single Sensillum Recordings (SSR)

We performed recordings with tungsten microelectrodes using standard equipment (IDAC-4, Syntech, Kirchzarten, Germany) and well-established experimental protocols (Andersson et al. 2009). For SSR, beetles from a newly established laboratory rearing (generations 1–2) were used. Responses were recorded and calculated with the Syntech software Autospike 3.0.

### Statistical Analyses

Chemical data were analysed with SPSS version 11.0.0, where differences between proportions of compounds in MeJ-treated and untreated bark were tested by one-way ANOVA. In order to fit the assumptions of normality and homogeneity of variances, data on proportions (*p*) were transformed by arc sin √*p*, where needed.

## Results

### Changes in Odor Profiles of Felled Trees over Time and in Stress-Induced Bark

**Fig. 1:**
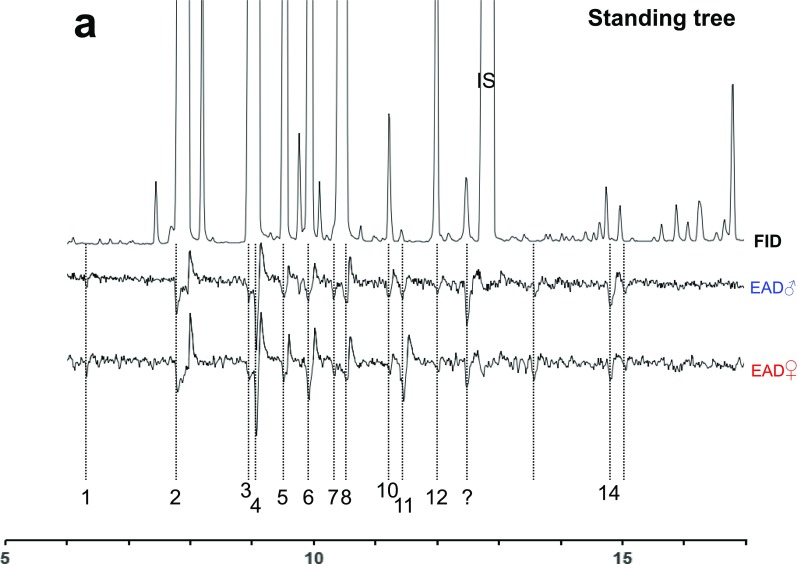

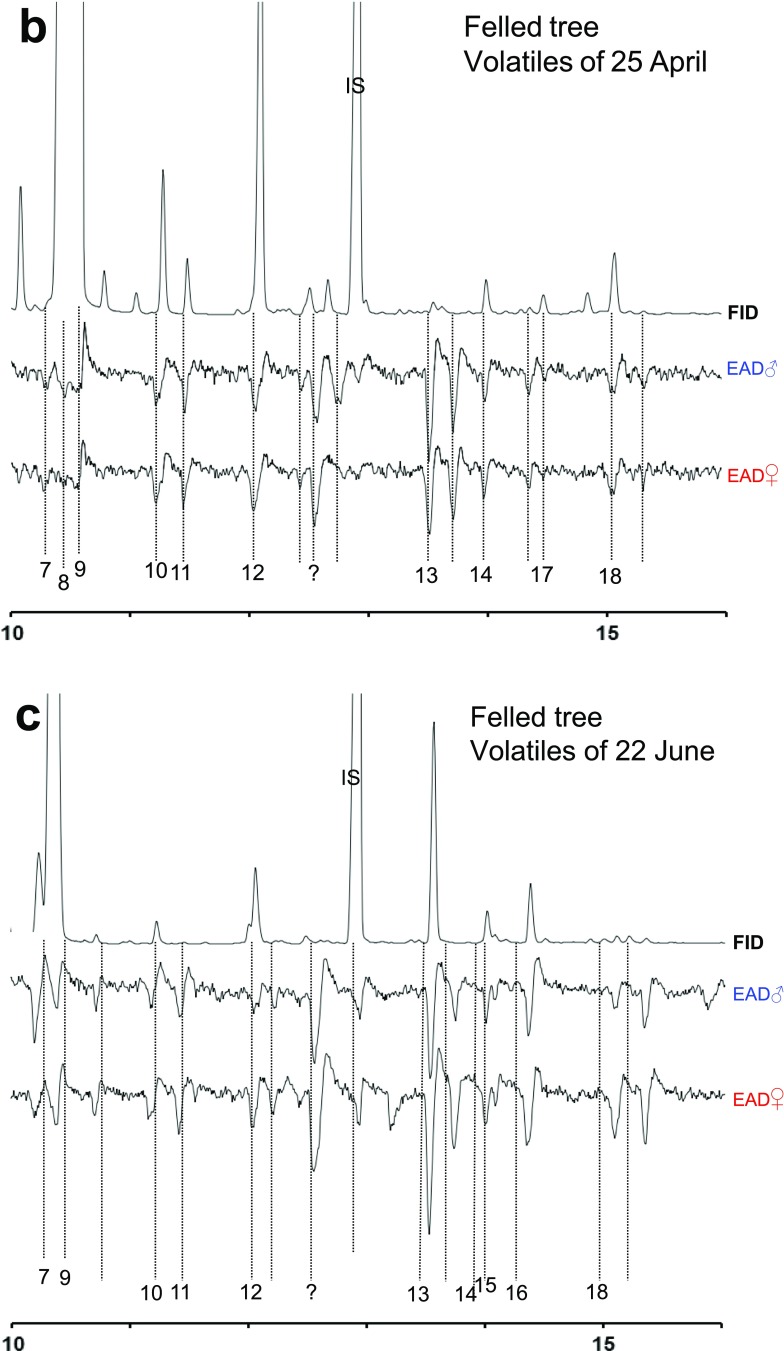
Representative examples of GC-EAD recordings (total 170 runs) from male and female *I. typographus* tested on natural volatiles collected from intact bark from standing or felled trees. **a** Example for the entire chromatogram from a sun exposed standing tree. Upper signal trace is FID signal and the two lower traces are corresponding recordings of antennal electric potentials of one male and one female beetle. **b** Volatiles from a felled tree sampled at the day of first attack (25 April) and **c** late in June. The graphs in **b** and **c** show only the part of the chromatogram where oxygenated compounds elute on a HP-5MS column. 1) styrene 2) α-pinene 3) sabinene 4) β-pinene 5) myrcene 6) 3-carene 7) *p*-cymene 8) limonene 9) 1,8-cineole (1,3,3-trimethyl-2-oxabicyclo[2.2.2]octane) 10) γ-terpinene 11) 4-thujanol; (synonym sabinene hydrate, 5-isopropyl-2-methylbicyclo[3.1.0]hexan-2-ol) 12) terpinolene 13) camphor (1,7,7-trimethylbicyclo[2.2.1]heptan-2-one) 14) pinocamphone (2,6,6-trimethylbicyclo[3.1.1]heptan-3-one) 15) pinocarvone (6,6-dimethyl-2-methylenebicyclo[3.1.1]heptan-3-one) 16) isopinocamphone (2,6,6-trimethylbicyclo [3.1.1]heptan-3-one) 17) 4-terpineol (1-isopropyl-4-methyl-3-cyclohexen-1-ol) 18) 4-allylanisole (estragole). IS) Internal quantification standard, heptyl acetate.?) Unidentified compound; Responses at the elution time of the internal standard are due to an unidentified co-eluting compound. Most recordings showed no responses to the internal standard. One strong response in some samples correlates well to nonanal, but response to synthetic nonanal could neither be observed in EAD- nor in SSR-recordings

The total 12 trees felled at the end of winter and subjected to aeration throughout the beetle flight season showed a clear trend of terpene oxygenation over time. The pattern was very consistent and strongly significant both at the group level and for each individual compound, as detailed in Table 1: Importantly, the group of 10 monoterpene hydrocarbons decreased during the sampling time (*r*_*S*=_ − 0.54**). In contrast, the group of oxygenated compounds (including the arenes estragole and styrene), increased in amount (*r*_*S*=_ + 0.51**). Standing trees showed low and shifting trends of oxidation (N.S., Table 1). Examples of chromatograms are shown in Fig. 1.

**Table 1:** Correlations (*r*_S_, Spearman’s rho correlation coefficient) of proportions of total release of all analyzed compounds with the factors sampling date, temperature at sampling, and attack density of *Ips typographus* (* and ** indicate correlation is significant (two-tailed) at the 0.05 level, or at the 0.01 level, respectively; positive *r*_S_: proportion of compound is rising with factor; negative *r*_S_: proportion of compound is declining with factor; − indicates compound was not detected in standing trees)

Compound classes	Sampling date (Julian date)		Temperature (°C)		Attack density
	Felled (*N* = 60)	Standing (*N* = 13)	Felled (*N* = 56)	Standing (*N* = 7)	Felled (*N* = 60)
Monoterpene hydrocarbons	−0.54**	−0.04	−0.44**	0.32	0.37**
Oxygenated terpenes	0.51**	−0.33	0.41**	0.10	−0.38**
Aromatic hydrocarbons					
Styrene	0.71**	0.52	0.39**	−0.64	0.57**
*p-*Cymene	0.16	−0.33	0.17	0.54	−0.05
Monoterpene hydrocarbons					
(±)-α-Pinene	0.31*	−0.18	0.17	−0.66	0.17
(±)-Camphene	0.16	0.34	−0.09	−0.03	−0.08
Sabinene & β-Pinene^a^	0.27*	0.37	0.22	0.13	0.20
Myrcene	−0.32*	−0.11	−0.18	−0.54	−0.15
3-Carene	−0.34**	−0.07	−0.14	−0.02	−0.33
(±)-Limonene & (±)-β-Phellandrene ^a^	−0.55**	−0.56*	−0.36**	0.17	−0.39**
Terpinolene	−0.54**	−0.15	−0.31	−0.22	−0.45**
γ-Terpinene	−0.61**	−0.33	−0.32	0.02	−0.52**
Oxygenated compounds					
4*-*Thujanol	0.21	–	0.12	–	0.22
1,8-Cineole	0.63**	−0.04	0.63**	0.09	0.44**
(±)-Camphor	0.50**	0.00	0.33*	0.43	0.30
Pinocamphone	0.57**	0.23	0.40**	0.09	0.46**
Pinocarvone	0.56**	0.41	0.28*	−0.43	0.41**
(±)-Terpinen-4-ol	0.08	−0.35	0.14	0.30	0.12
α-Terpineol	0.05	−0.33	0.03	0.30	0.09
Estragole	0.36**	0.00	0.32*	0.65	0.31*
Verbenone	0.64**	–	0.31*	–	0.55**

^a^These compound pairs co-eluted

**Fig. 2:**
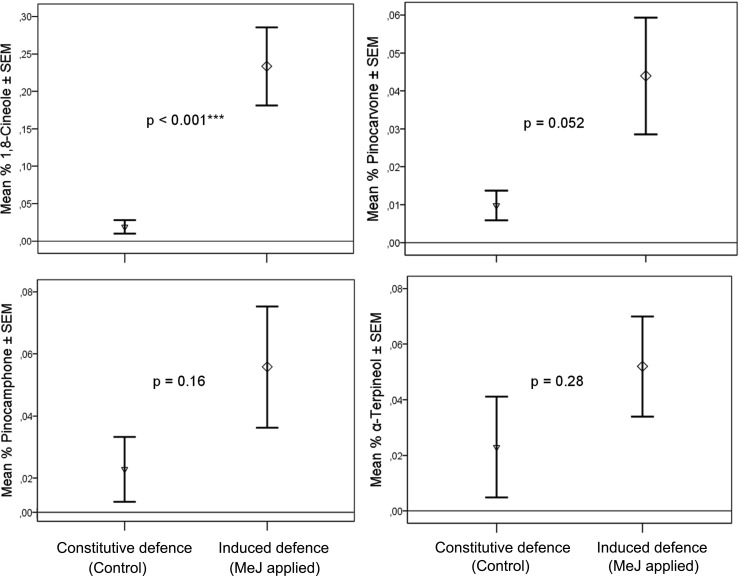
Induction of four of six physiologically active metabolites in trees locally treated with the phytohormone methyl jasmonate (MeJ). Two metabolites showed no change (data not shown). Proportions of oxygenated monoterpenes in bark of untreated (control) compared to MeJ-treated (MeJ) bark in seven healthy standing trees. Trees were treated with a local MeJ application and bark sampled nearby (Schiebe et al. 2012). In each graph centre *p*-values from ANOVA are given for the difference of sample means of treated/untreated trees

The bark that had been stress-induced with MeJ had overall more 1,8-cineole, pinocarvone, pinocamphone, and α-terpineol. However, differences were significant only for 1,8-cineole and close to significant for pinocarvone, with pinocamphone and α-terpineol following the same trend (Fig. 2). The proportions of camphor, 4-terpineol, and the aromatic compounds styrene and 4-allylanisole were not influenced or slightly decreased by MeJ-treatment (not shown).

Natural beetle attacks promoted similar stress-induced release of volatiles. The release was higher in mid crown samples from cut trees (cut in February and April) compared to five mid crown samples from healthy standing trees (ANOVA and Dunnett’s T3 Post Hoc *P* < 0.005**). The release from the mid crown of standing trees under newly-started natural attacks, however, equaled that of cut trees in late April. All of the six successfully naturally attacked standing trees had release rates between 250 and 3100 μg per measured area (≈ 5 dm^2^ / 4 h), but only two of seven unattacked trees had rates >50 μg.

Release of all nine oxygenated compounds increased with attack density on felled trees (Table 1), five of these in particular, i.e. 1,8-cineole, pinocamphone, pinocarvone, and verbenone (*P* < 0.01**, Table 1).

### GC-EAD Analysis of Spruce Volatile Samples

A total of 170 GC-EAD runs were made for 19 different volatile samples, from seven felled and two standing spruce trees collected at ten different time points during April to June, using two GC columns of different polarity. Three examples of beetle antennal responses are shown in Fig. 1: A complete volatile sample chromatogram from one standing tree (Fig. 1a) and chromatogram sections showing oxygenated compounds from a tree two times after felling (Fig. 1b and c). No clear differences in the response from the two sexes were noted.

The strongest EAD responses compared to peak size in the chromatograms were elicited by five oxygenated monoterpenes (cyclic ether, alcohols, and ketones): 1,8-cineole, 4-thujanol (sabinene hydrate), 4-terpineol, camphor, and pinocarvone. Strong responses were also recorded to two peaks identified as pinocamphone and isopinocamphone. Another very strong EAD response was regularly recorded prior to the internal standard heptyl acetate. This response coincided exactly with a peak chemically identified as nonanal in some samples, but no physiological response was elicited by synthetic nonanal. In addition, no OSNs tested in SSR studies responded to nonanal. A phenylpropanoid, 4-allylanisole (estragole), elicited clear EAD responses in most runs. A compound reproducibly present in very small amount that elicited strong antennal activity was identified as styrene. Responses to synthetic blends (not shown) also included five known oxygenated host compounds that, however, were not clearly identified in our natural samples; (±)-α-terpineol, (±)-carvone, (±)-ipsenol, (±)-ipsdienol, and ipsdienone. Highly repeatable responses were also elicited by four main terpene hydrocarbon constituents of the spruce bark bouquet: α-pinene, β-pinene, myrcene and 3-carene. No responses were obtained to (±)-sabinene. In contrast, three minor hydrocarbon constituents: *p*-cymene, γ-terpinene and terpinolene, generally elicited comparably stronger responses than the major host constituents (the pinenes, myrcene, 3-carene, limonene and β-phellandrene).

### Single-Sensillum Recordings

In all we tested 35 compounds. The spontaneous activity of two OSNs within the same sensillum could generally be separated by amplitude in SSR. Most of the responses were recorded from the OSN with the largest amplitude (the A-neuron) and only in few recordings did both the A- and the B-OSN respond to tested compounds.

**Fig. 3:**
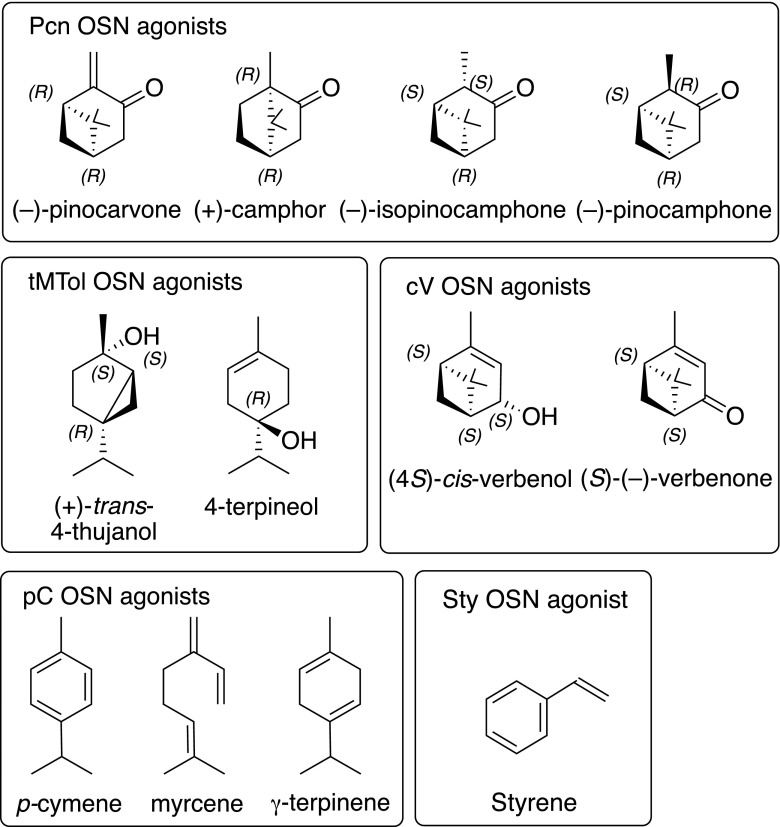
Structures of electrophysiologically active compounds identified for the various OSN classes found. The stereochemistry assignments of the camphone stereoisomers are tentative, based on structural resemblance with (+)-camphor and (-)-pinocarvone in the Pcn OSN class

Six classes of OSN are characterized, including three new ones (Table 2, Fig. 3), based on their response spectra as detailed below.

**Fig. 4:**
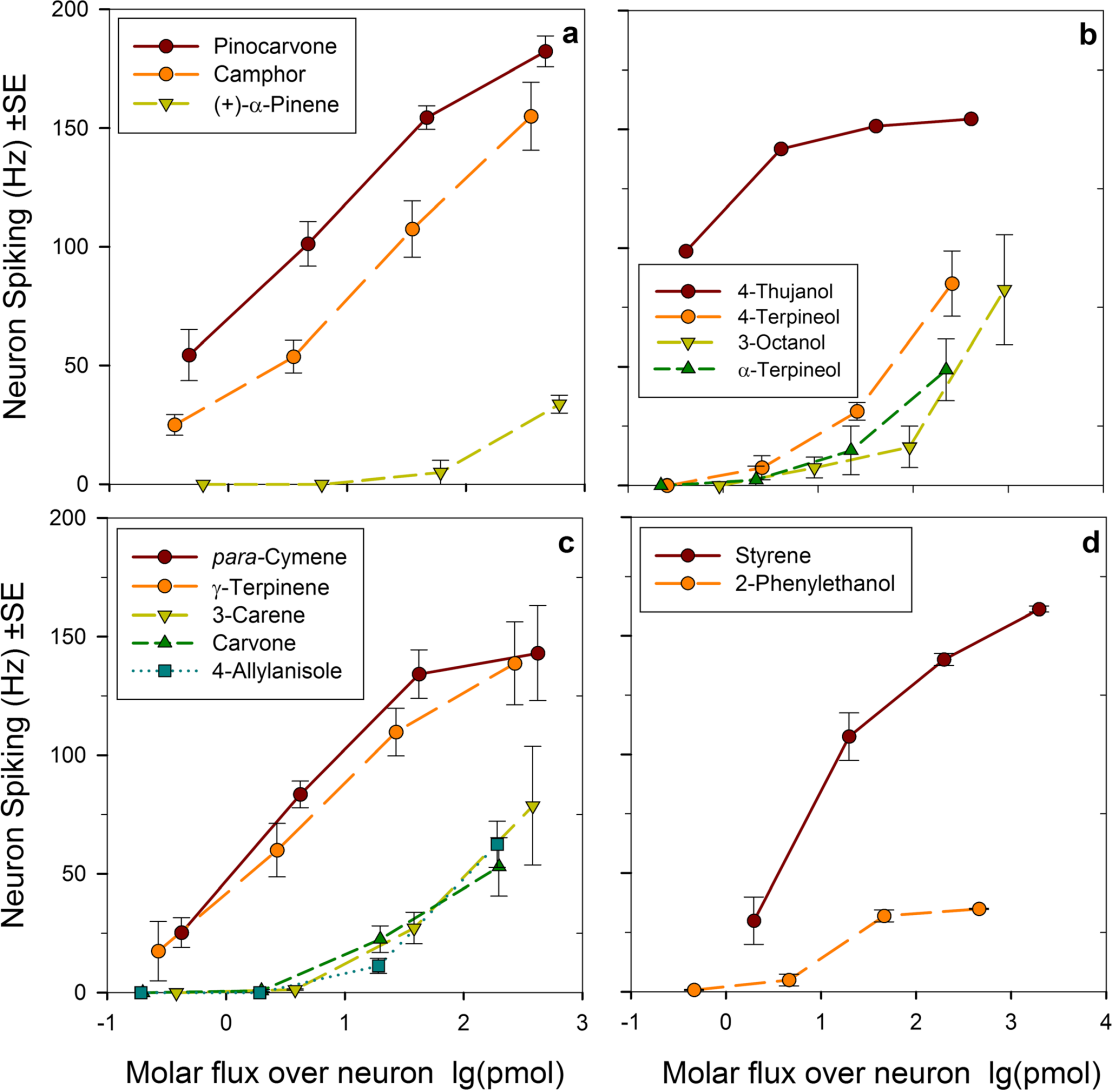
Volatility corrected dose-response of OSN-classes **a** Pinocarvone and other ketones (Pcn). **b** tertiary monoterpene alcohols (tMTol), **c***p*-cymene derivatives (pC), and **d** phenyl ethane derivatives (Sty). Odorant doses (x-axis) correspond to the theoretical flux of molecules (in pmol) from the stimulus cartridge during a stimulus puff, compensating for the differential volatility and affinity to paraffin oil solvent between chemicals (Andersson et al. 2012). Error bars indicate SEM (*n* = 5, *n*= 2 for styrene)

**Fig. 5:**
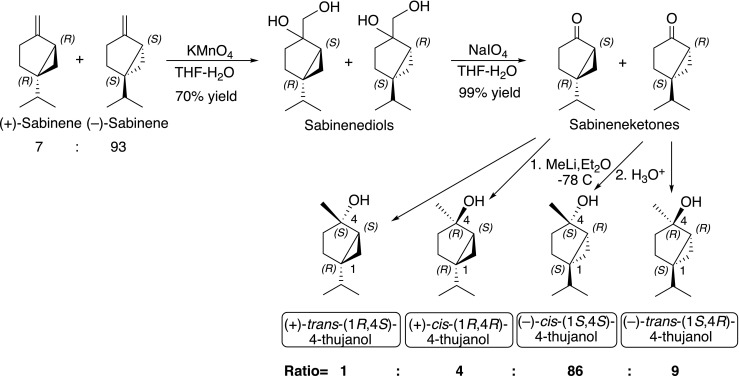
Regioselective synthesis of all four stereoisomers of 4-thujanol (sabinene hydrate) from sabinene

1. The New OSN for Pinocarvone and other Ketones (Pcn) (Table 2, Fig. 3, Fig. 4a) This OSN type was found in 14 sensilla subjected to SSR. Pinocarvone elicited the strongest response but was not available from the start of the experiments and was included in the panel only in the last three recordings. Before then, (±)-camphor was the most active ligand. The Pcn-OSN is very likely tuned to several ketones with similar configuration (Fig. 3) as putative responses were found in GC-EAD recordings also to both isopinocamphone and pinocamphone, but neither of these compounds was available in synthetic form for the SSR study. Furthermore, (+)-α-pinene and (−)-β-pinene regularly elicited weak responses from this OSN-type (Table 2, Fig. 4a).
2. A New OSN for Tertiary Monoterpene Alcohols (tMTol) (Table 2, Fig. 3, Fig. 4b) The most active ligand for this OSN type was 4-thujanol. Unfortunately, we could only test this compound as a synthetic standard on one antenna in SSR due to its late identification in GC-EAD recordings. The 4-thujanol peak in bark headspace samples regularly elicited strong responses in GC-EAD and co-eluted with the synthetic compound. The syntheses and enantioselective analysis (Fig. 5) of the four stereoisomers of 4-thujanol revealed that the commercial Aldrich-Fluka sample used by us consisted of 98% *trans*-(+)-isomer. The strong beetle response is therefore, with highest probability, to *trans*-(+)-4-thujanol. This neuron-type responded with modest responses to (±)-4-terpineol, (±)-3-octanol and (±)-α-terpineol (Table 2, Fig. 4b).
3. The Known *p*-Cymene OSN (pC) (Table 2, Fig. 3, Fig. 4c) Apart from *p*-cymene, this OSN responded similarly strong also to γ-terpinene, a host monoterpene with similar structure (Fig. 3), and with medium strength to the monoterpene hydrocarbons (±)-limonene and terpinolene and to the oxygenated compounds (±)-carvone and *p*-allylanisole. 3-Carene also gave a medium strength response, which is somewhat surprising as the structure is not very closely related to the ones of cymene or menthene derivatives (Fig. 4c).
4. Compounds Active on the New Styrene OSN (Sty) (Table 2, Fig. 3, Fig. 4d) Several times during earlier screening, before styrene could be included in the panel, a neuron responding modestly and exclusively to 2-phenylethanol was found. After chemical identification of the early eluted and strong EAD-active peak, styrene was included in later test panel On the antennae of two males an OSN-type was found that responded with a strong phasic response to styrene and with a pronounced tonic response of medium strength to hydrated styrene; 2-phenylethanol (Fig. 4d). None of the other compounds in the test panel elicited any response in the styrene OSN.
5. Compounds active on the known pheromone OSN (cV) and the myrcene OSN (My) (Table 2, Fig. 3) The OSN (cV) attuned to the semiochemicals *cis*-verbenol and verbenone was found two times during this investigation despite the fact that the pheromone OSN region of the antennae was avoided (Table 2). The myrcene-specific My-OSN was found only once in one male.

**Table 2:** Test panel of compounds for single sensillum recordings from *I. typographus* OSN*s*

Compound	Chemical source Purity %	Substance origin	Responses in OSN classes^1^					
			Pcn	tMTol	pC^#^	cV^#^	My^#^	Sty
Hydrocarbons								
Styrene *	Sigma; >99	Host						**••••** ^2^
(+)-α-Pinene	Janssens Chim; 98	Host	**••**		(••)			
(–)-β-Pinene *	Fluka; 92	Host	**•••**					
Myrcene	Fluka; 95	Host			(••••)		••••	
(+)-3-Carene	Aldrich; 93	Host			**•••**			
*para-*Cymene	Acros; >99	Host			**••••**			
(±)-Limonene	Fluka; 98	Host			**•••**			
γ-Terpinene	Fluka; 99	Host			**••••**			
Terpinolene	Fluka; 97	Host		(•)	(•••)			
Alcohols								
(+)-Borneol	Sigma 98	Host	(•)		(•••)	(•)		
1-Hexanol	Fluka; >99	NHV						
2-Methyl-3-buten-2-ol	Aldrich; >97	Ph						
*E*-Myrcenol	SciTech Ltd,; 95	Ph						
(±)-Ipsenol	Bedoukian; 95	Ph		(•)				
(±)-Ipsdienol	Bedoukian; 95	Ph						
(±)-3-Octanol	Acros; >99	NHV/Fungi		**•••**				
2-Phenylethanol	VWR Int.;>99	Ph	(•)	(•)		(•)	(•)	••
(±)-4-Terpineol	Fluka; >99	Host	(•)	**•••**	(•)		(•••)	
(±)-α-Terpineol	Aldrich; 98	Host		**••**				
(+)-*trans*-4-Thujanol	Aldrich; >98	Host	(•)	(•••••)				
(–)-*cis*-Verbenol	Borregaard; 95	Ph				**••••**		
Ketones								
(±)-Camphor	Fluka; >95	Host	**••••**			(•••)		
(±)-Carvone	Acros; >98	Host			**•••**	(•)		
Pinocarvone *	Synergy C.; 51^a^	Host	(**•••••**)					
(–)-Verbenone	Fluka; >99	Host				•••		
(±)-α-Thujone	Sigma; >97	Conifer	(•)	(•)				
Other oxygenated								
(+)-*exo*-Brevicomin	W.F.^b^; 99	Ph						
(±)-Chalcogran	Celamerck; 93	Ph		(•)				
(±)-*trans*-Conophthorin	W.F.; 95	NHV/Fungi		(••••)	(•)			
1,8-Cineole	Aldrich; >99	Host		(•)	(•)			
4-Allylanisole	Acros; 98	Host	(•••)		**•••**			
Nonanal	Fluka; >95	Host						
No. of cells found								
Male			5	5	7	2	1	2
Female			9	2	0	0	0	0
Total			14	7	7	2	1	2

^1)^Olfactory sensory neuron (OSN) classes are defined according their primary responses. Pcn: Pinocarvone, tMTol: tert. monoterpenols, pC: *p*-Cymene, cV: *cis*-Verbenol, My: Myrcene, Sty: Styrene

^2)^responses: • = 30 – 50 Hz, **••** = 51 – 75 Hz, ••• = 76 – 100 Hz, •••• = 101 – 150 Hz, ••••• >150 Hz. Responses in parentheses were found only occasionally or tested few times. #) OSN – class according to Andersson et al. (2009)

^a^Concentrated extract kindly provided by Synergy Semiochemicals Corp., Burnaby, Canada

^b^Wittko Francke, University of Hamburg, Germany *) compounds that were added to the panel late in the study

### Synthesis of 4-Thujanol Stereoisomers

The double bond in (−)-sabinene (86% ee, Aldrich) was oxidized to the corresponding diol which was subsequently cleaved to sabinaketone (Fig. 5). After the reaction with methyl lithium a reaction mixture of all four 4-thujanol stereoisomers was formed that could be analytically separated on an enantioselective GC-column (β-cyclodextrin phase). Based on the ratio of stereoisomers in the original sabinene (93/7 (−)/(+)), it was easy to differentiate the (+)-(1*R*)- and (−)-(1*S*)-forms of sabinaketone from each other. The methyl lithium addition to the carbonyl of sabinaketone proceeded stereoselectively i.e. the methyl group was added 10 times more frequent from the less sterically hindered side giving rise to a 10:1 excess of *cis*-forms over the corresponding *trans*-forms (see Fig. 5). After comparison of the NMR of the *cis*-forms with the *trans*-isomer from Aldrich (Baeckstrom et al. 1996) the major product could be assigned to (−)-*cis*-4-thujanol both from the synthesis sequence and regioselective consideration, as well as literature NMR data (Baeckstrom et al. 1996). The four isomers were injected on a β-cyclodextrin GC-column and the commercial 4-thujanol stereoisomer obtained from Sigma-Aldrich co-eluted with the (+)-*trans*-4-thujanol peak.

The elution order correlated with the one by Marriott et al. (2001) and was further confirmed by analysis of marjoram, which according to Larkov et al. (2005) mainly produce the (+)-forms of sabinene hydrate (Larkov et al. 2005; Marriott et al. 2001). Hence, our unambiguous assignment is that the pure stereoisomer provided by Sigma-Aldrich and tested by us is (+)-*trans*-(1*R,*4*S,*5*S*)-4-thujanol.

## Discussion

To a bark beetle it is of utmost importance to obtain information regarding the physiological status of a potential host tree. This information is vital both for initial survival and ultimately for reproduction. The final decision to enter a tree or not may be taken first after landing, guided by close range olfactory or gustatory cues. Stress-related host compounds, often of anti-feedant or anti-attractant activity, may be of significant importance for this decision. We found that the *I. typographus* olfactory system, both at EAD and single neuron levels, displays high sensitivity to stress-related host compounds. GC-EAD analyses regularly revealed clear responses to peaks too small to identify by GC-MS if not known and identified earlier. By the use of selected ion chromatograms in GC-MS, the presence of known and earlier identified compounds could be confirmed.

We observed a number of new EAD responses to host compounds, of which most were verified by recording responses to synthetic compounds and further by SSR. By SSR, we were able to identify three additional compounds eliciting OSN-responses. Six of these 10 compounds are oxygenated monoterpenes. Several other unidentified responses were recorded in the retention time range where most oxygenated monoterpenes elute. One of the compounds eliciting the strongest response was identified as (+)-*trans*-4-thujanol, which also gave rise to the strongest response from one specific OSN-type (tMTol). This compound was found only in trace amounts in most volatile samples, and could only be positively identified by comparison with synthesized material. Other minor compounds eliciting strong regular responses, like camphor, pinocarvone, and pinocamphone could be identified and quantified in both volatile and bark samples. These findings add information to previous hypotheses of how *Ips typographus* beetles chose their hosts.

We observed that oxygenated compounds increased both over time in felled trees and after induction of defence by MeJA application and that some of these compounds were GC-EAD active.

Oxygenated monoterpenes (MTO) can be derived by autoxidation but are mainly produced by microbial oxygenation of hydrocarbon precursors (Birgersson et al. 1984; Hunt et al. 1989; Leufvén et al. 1984; Lindmark-Henriksson et al. 2004). Compounds such as linalool, borneol, and α-terpineol can also be products of induced monoterpene synthase activity in the tree (e.g., Martin et al. 2003). Many studies assessing conifer defense responses concurrently found pronounced quantitative changes of terpene hydrocarbons but no clear changes in relative composition of oleoresin constituents (Boone et al. 2011; Leufvén and Birgersson 1987; Martin et al. 2002; Raffa and Berryman 1982a; Raffa and Berryman 1982b; Raffa and Smalley 1995; Schiebe et al. 2012; Zhao et al. 2010). In contrast, the proportional changes found for oxygenated monoterpenes in MeJ induced bark in the present study, and previously shown for MeJ treated foliage of *P. abies* (Martin et al. 2003), make these compounds plausible candidates as indicators for host resistance. Indeed, the compound with strongest increase after MeJ induction in our study, 1,8-cineole, is already a firmly established attractant inhibitor (Andersson et al. 2010, 2011) with an effect similar to that of verbenone (Binyameen et al. 2014), when tested with *I. typographus* aggregation pheromone.

Bark beetle responses to monoterpene hydrocarbons at much higher release rates may function more as habitat signals in the discrimination between non-host and host habitats (Zhang and Schlyter 2003; Zhang and Schlyter 2004). They also might modulate their behavioral responses to MTOs, as well as to pheromones (Erbilgin et al. 2007; Hulcr et al. 2006; Jakus and Blazenec 2003).

The phenylpropanoid 4-allyanisole also showed medium to high activity in both GC-EAD and SSR-studies. Chemical analyses of volatiles and bark content in this study did not show any proportional changes as for oxygenated terpenes. In a previous study it did, however, increase in parallel to the terpenoids after MeJ-treatment (Schiebe et al. 2012). This compound is a known attractant inhibitor for *D. ponderosae* and *I. pini* (Hayes and Strom 1994).

The most common OSN type encountered, (Pcn), responds to α-methylated monoterpene ketones, e.g. camphor and pinocarvone. Interestingly, this ubiquitous OSN type was also found in the major bark beetle predator *Thanasimus formicarius* (Tømmerås and Mustaparta 1987).

Styrene elicited a strong phasic response in a specific OSN-type. The hydrated analogue, 2-phenylethanol, elicited a somewhat weaker response from the same neuron, but with a pronounced tonic component. 2-Phenylethanol has been proposed as a pheromone component of *I. typographus*, when it was found in the hindguts of males (Birgersson et al. 1984), but no biological activity was established (Schlyter et al. 1987). Male beetles of several *Ips* bark beetles do produce 2-phenylethanol, while females do not (Birgersson, unpubl.) *Ips pini* (Say) has been shown to produce 2-phenylethanol and toluene from phenylalanine when boring into fresh pine logs (Gries et al. 1990). In a recent study, styrene has been shown to be produced by *Penicillium expansum*, a fungus isolated from the pine weevil *Hylobius abietis* (L.) (Azeem et al. 2013), and it has also been shown to increase significantly in logs infested by *I. typographus* (Pettersson and Boland 2003). In our volatile samples from felled trees it was found more often in very low amounts also before colonization started and in one standing tree, but was found in higher frequency in samples from felled trees later in time. The response to styrene can possibly be interpreted as a signal indicating fungal activity in old hosts, competing galleries or a successful establishment of symbiotic fungi.

The strong sensitivity to compounds present at low levels may have evolved to detect small shifts in the host odor bouquet as an important indicator of host state for attacking beetles, possibly detectable after landing but before committing to enter the bark. It has been reported that *trans*-4-thujanol is found in higher amounts in young spruce trees in Lithuania, and is therefore a tentative host-age indicator (Blažytė-Čereškienė et al. 2016). Such a function might range from indicating a dangerous, defending host, or a host with impaired defenses, to a host unsuitable due to degradation or to intra- or interspecific competition. Both absolute and relative amounts in the odor bouquet encountered by the beetles might be essential to elicit the adequate behavior. In other studies, we recently found *trans*-4-thujanol to have an activity similar to 1,8-cineole in reducing the attractiveness of synthetic pheromone in the field (Jirošová et al. unpublished).

Our combined chemical and neurophysiological analyses strongly indicate that small amounts of oxygenated compounds are candidate indicators for host stress and should, as such, be important for the evaluation of host quality and vitality by the beetles. Behavioral studies are ongoing to assess the effect of individual stress compounds and their blends in conjunction with the volatiles emitted by the symbiotic fungi associated with the beetles, as the next step in understanding this intricate system. The effect of non-volatiles (e.g., non-volatile phenolics and lipids) as gustatory cues of host acceptance deserve attention and should be the subject to future studies. Our results add to the basic knowledge of chemical cues of host selection in *I. typographus* and may be applicable also to other tree-killing bark beetles.
